# A combination of two methods for evaluating the usability of a hospital information system

**DOI:** 10.1186/s12911-020-1083-6

**Published:** 2020-05-04

**Authors:** Reza Khajouei, Fatemeh Farahani

**Affiliations:** Department of Health Information Sciences, Faculty of Management and Medical Information Sciences, Kerman University of Medical Sciences, Kerman, Iran

**Keywords:** Usability evaluation, Human-computer interaction, Heuristic evaluation, Think aloud, Hospital information system

## Abstract

**Background:**

None of the evaluation methods can identify all the usability problems of information systems. So far, no study has sufficiently investigated the potential of a combination of these methods to identify usability problems. The present study aimed at examining the potential for combining two commonly utilized user-based and expert-based methods to evaluate the usability of a hospital information system.

**Methods:**

Think aloud (TA) and Heuristic evaluation (HE) methods were used to identify the usability problems of two subsystems of the Social Security Electronic System in Iran. To this end, the problems were categorized into five groups based on ISO-Nielsen usability attributes. The Chi-square test was applied to compare the intended methods based on the total number of problems and the number of problems within each group, followed by utilizing the Mann-Whitney U test to compare the mean severity scores of these methods.

**Results:**

The evaluation by combining these methods yielded 423 problems of which 75% varied between the methods. The two methods were significantly different in terms of the total number of problems, the number of problems in each usability group, and the mean severity of two satisfaction and efficiency attributes (*P* < 0.05). However, no significant difference was observed between the two methods based on the mean severity of problems and severity scores related to three usability attributes i.e., effectiveness, learnability, and error prevention (*P* > 0.05). In addition, the mean severity of problems identified by each method was at the “Major” level.

**Conclusion:**

Based on the results, although the mean severity scores of the identified problems were not significantly different, these methods identify heterogeneous problems. HE mainly identifies problems related to satisfaction, learnability, and error prevention while TA detects problems related to effectiveness and efficiency attributes. Therefore, using a combination of these two methods can identify a wider range of usability problems.

## Background

Usability evaluation is considered critical for successful implementation and optimization of an information system. According to the International Standard Organization (ISO) [1] usability means the extent to which special users can utilize a product to achieve specific goals in particular environments by considering effectiveness, efficiency, and users’ satisfaction. Nielsen [2] defines usability as “a quality attribute which assesses how easy user interfaces are to use”. According to Nielsen, usability is defined by five components including learnability, efficiency, memorability, error prevention, and user satisfaction. In addition, usability evaluation aims to improve a software system by identifying its usability problems and prioritizing them based on their impact on the users. In the field of healthcare, usability concerns the understandability, learnability, acceptability, attractiveness, usefulness, and performance of Healthcare Information Systems. Further, it evaluates how easy these systems are operated by users and to what extent support them to provide effective healthcare services to patients.

Some usability evaluation studies [3–5] focused on identifying usability problems in health information systems which affect the users and healthcare organizations. During the past few decades, developers have emphasized the evaluation of healthcare information systems in order to support their users in healthcare organizations since usability evaluation is regarded as an important component in the development process of an information system.

A large number of users with different backgrounds interact with the systems in clinical and administrative environments. Given the limited time of the health care providers, especially nurses, to learn a new information system, on the one hand, and the high cost of training on the other, an appropriate assessment method is required to determine the usability of such a system and to help reducing the time and cost of training [6].

In general, user-based testing and expert-based inspection methods are the two main types of usability evaluation methods [6]. Different usability methods play unique roles in detecting the problems [7, 8] and each method has its advantages and disadvantages. For example, user-based methods mostly detect special problems which prevent users from performing tasks while expert-based methods often identify general user interface problems. Think aloud (TA) and Heuristic Evaluation (HE) are the most common types of these two methods, respectively [6, 9]. TA method, which originated from the field of cognitive psychology, encourages users to express out loud what they are looking at, thinking, doing, and feeling, as they perform tasks [10]. TA is considered as the golden standard of usability evaluation methods, since it provides an in-depth insight into the problems during the user-system interaction [6]. Data obtained from this type of evaluation provides a valuable opportunity for identifying specific problems which the users experience during their workflow [11]. HE is regarded as an informal usability inspection technique in which experts evaluate whether user interface elements of a system adhere to a set of usability principles known as heuristics [12] . Furthermore, HE is a simple and cost-effective method which identifies “Minor” and “Major” problems in a system user interface. Moreover, both methods can be employed in the formative and summative evaluation of a system [6]. Given the potential of each of the user-based and expert- based methods for identifying specific problems in a system, the previous studies have emphasized on using a combination of different evaluation methods [6, 13] . Additionally, according to some studies [9, 14–16] the combination of HE and TA can pave the way for designing user interfaces which are appropriate for novice and low experienced users. However, the previous studies [9, 14–17] that utilized TA and HE methods neither combined the results of both methods nor applied statistical analysis for comparing the methods. In addition, they focused on evaluating a single system with a small sample of usability evaluators or participants. Conducting a study with a sufficient number of evaluators or users and utilizing both quantitative and qualitative analysis can reveal the potential of each method and a combination of methods for detecting different types of usability problems.

The Social Security Electronic System (SSES) is considered as one of the widely used Hospital Information Systems in Iran, which has recently been implemented in all hospitals affiliated with Social Security Organization (SSO). According to a previous study [18], users were not completely satisfied with this information system. Since satisfaction is regarded as one of the main usability components, usability evaluation of this system can reveal the problems diminishing it and other usability components such as effectiveness. Hence, the results of usability evaluation by the above-mentioned methods can help improve the acceptance of the information system by the users in their administrative and clinical environments, resulting in fulfilling the main goals of the SSO such as improving the health and safety of the patients.Previous similar studies in Iran used a standard checklist or questionnaire [19, 20], the TA followed by a questionnaire [21], and the HE method [22, 23] to evaluate the usability of health information systems. To our knowledge, so far no evaluation study has specifically investigated the effectiveness of a combination of user-based and expert-based usability methods worldwide. Accordingly, the present study sought to examine the potential of combining the TA and HE methods for evaluating two main administrative and clinical modules of the SSES (inpatient admission and nursing information systems). The results of the present study are expected to help the designers improve the design of user interfaces of health information systems.

## Methods

### The aim, design and setting of the study

The current study was conducted to evaluate the usability of the inpatient admission and nursing information modules of the SSES in Iran by combining the Think aloud and Heuristics evaluation methods in 2018.

This study was performed at Payambare-Aazam Hospital in Kerman, which is the largest social security institution in the southeast of Iran. This hospital is ranked sixth in terms of the number of beds among other social security institutions of Iran. The inpatient admissions module of the SSES is mainly used for admitting the inpatient, transferring patients from the emergency rooms to one of the inpatient wards, allocating patients to a clinical ward, providing different statistical reports, as well as managing files, insurance claims and patient discharges. The nursing information system of the SSES is utilized for procedures such as requesting laboratory tests, medications, and other para-clinical materials, as well as recording consultations, physician visits, and all clinical procedures. In the present study, the TA was performed on the nursing information system used for Intensive Care Unit (ICU).

### The characteristics of participants

The user interfaces of the two information systems were evaluated by eight medical informatics specialists who were trained in heuristics evaluation (HE). In addition, 18 senior nursing students and 17 undergraduate and postgraduate students in health information technology and medical informatics were invited as the potential users of the nursing information system and inpatient admission information system to participate in the TA tests. None of the participants had working experience with the nursing information and inpatient admission systems of the SSES. The TA tests were conducted in laboratory conditions and away from the actual clinical environment in order to preserve the patients’ safety.

### The description of materials

#### Heuristics evaluation

Eight evaluators independently examined the design of user interfaces related to both nursing information system and inpatient admission information system against 10 Nielsen principles [6] in three to four sessions. Each session lasted approximately two hours and the evaluators identified the violations of each principle as a usability problem and entered them into a list.

#### Think aloud

To perform the user testing, first, a number of scenarios which cover most of the user’s tasks were designed in consultation with the end-users and the heads of inpatient admission wards and nursing departments.

**Fig. 1:**
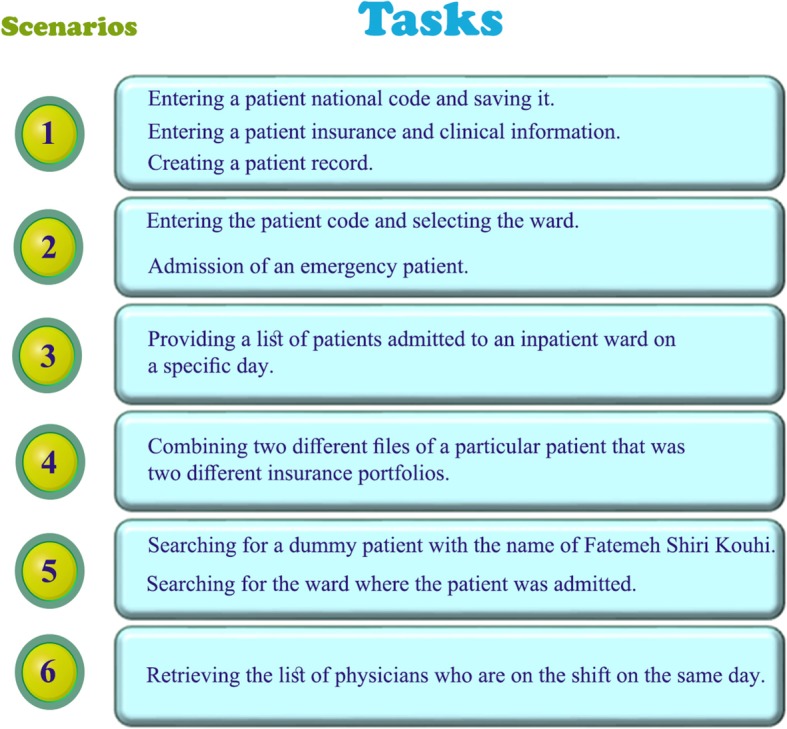
Six scenarios comprising 10 tasks in the admission department

**Fig. 2:**
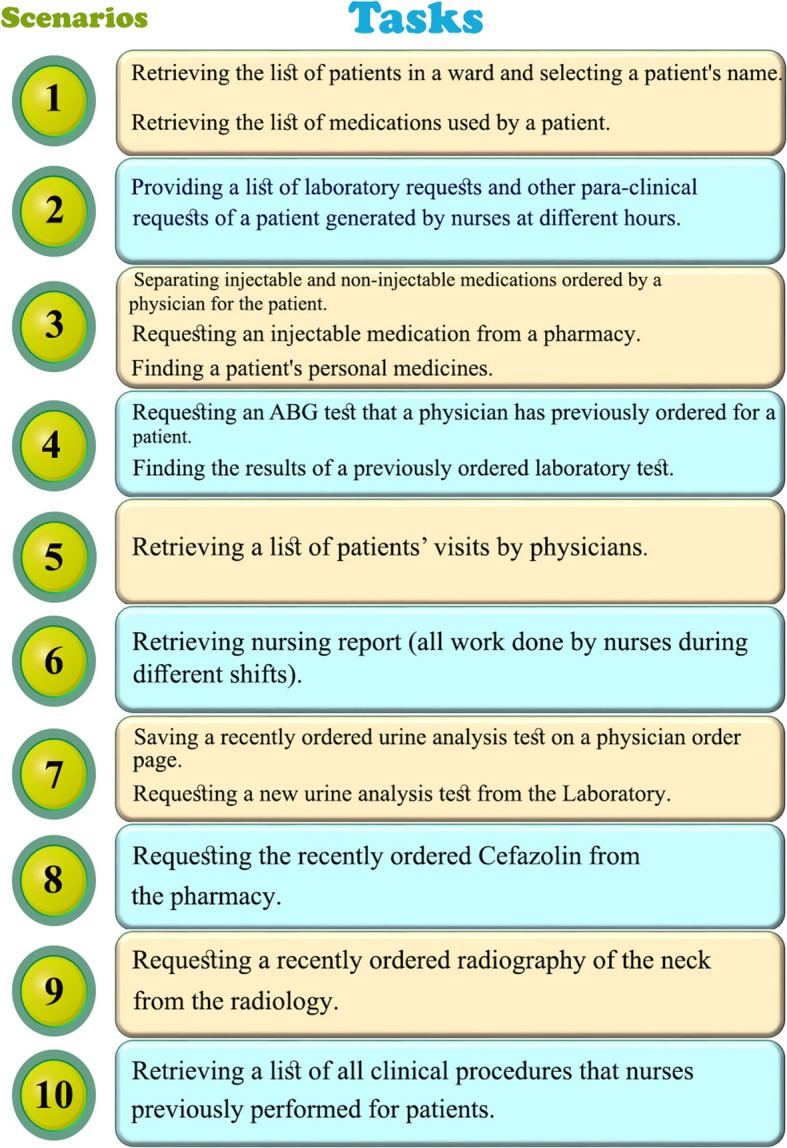
Ten scenarios containing 15 tasks in ICU

Figures 1 and 2 illustrate the six and ten most common scenarios used for evaluating the inpatient admission information system and the nursing information system, respectively.

Then, all interactions of users with the systems including their speech, gestures, and their actions on the screen were captured using Morae Recorder version 3.3 (TechSmith Corp.) in 35–45 min sessions. Next, eight independent evaluators reviewed all recordings, utilizing Morae Manager in order to identify the problems which the users encountered during their interaction with the systems. Then, these evaluators independently assigned a severity score ranging from 0 to 4 [24] (Table 1) to each identified problem based on three criteria proposed by Nielsen, including the frequency, impact, and persistence [25]. Finally, all problems were classified according to the combination of the six usability attributes proposed by ISO and Nielsen [1, 2], i.e., satisfaction, effectiveness, efficiency, learnability, memorability, and error prevention. It is worth mentioning that the memorability attribute was impossible to evaluate and thus was removed from the classification since the participants interacted with each system only once.

**Table 1:** The rate of problems based on their severity

Rate	Severity	Description
0	**No problem**	No usability problem;
1	**Cosmetic**	No need to be fixed unless extra time is available on the project;
2	**Minor**	Fixation should be given low priority;
3	**Major**	Important to fix thus it should be given high priority;
4	**Catastrophe**	Imperative to fix before releasing the product

### Data analysis and comparisons

#### Qualitative analysis

Duplicate problems were eliminated during three stages as follows. First, all evaluators met in two sessions to investigate the individual lists of problems identified by each method in each system (four lists of problems) and remove duplications within each list. Second, duplicate problems between the two lists of the identified problems by each method in two systems were eliminated in a session and a single list of problems for each method was obtained accordingly. At this stage, the problems were categorized into five groups of ISO-Nielsen usability attributes. Eventually, the duplicate problems between the lists of the two methods were removed, in order to integrate the problems identified by both methods, and the evaluators approved the final list of usability problems in a session.

#### Quantitative analysis

Data related to all three methods (i.e., TA, HE, and the combined method) were analyzed using SPSS, version 25 (SPSS Inc., Chicago, IL, USA). Further, the Chi-square test [26] was utilized to compare the total number of problems identified by TA and HE methods, as well as the number of problems categorized into different groups between the methods. Ultimately, the relationship between the mean severity scores of problems identified by TA and HE was evaluated using the Mann-Whitney U test since the distribution of the data was not normal.

## Results

Table 2 demonstrates the number of problems identified by TA, HE and a combination of these methods, as well as the number of similar problems between the two methods based on ISO-Nielsen usability attributes. As a result, 423 problems remained by removing duplicate problems. HE identified 268 problems in nursing information system and 180 problems in inpatient admission information system. The elimination of duplicates yielded 163 unique problems detected by HE. The number of identified problems using TA in nursing and patient admission information system were 72 and 88, respectively. After eliminating the duplicates between these two groups of problems, 127 unique problems were remained. Finally, forty-five problems were identically identified by both methods.

**Table 2:** The number of the identified problems per method and usability group

	Heuristics evaluation N (%)	Think aloud N (%)	Think aloud + Heuristics evaluation N (%)	Both Think aloud and Heuristics evaluation N (%)	Only Think aloud N (%)	Only Heuristics evaluation N (%)
Satisfaction	120 (44.6)	113 (43.46)	190 (45)	43 (40.5)	70 (45.45)	77 (47.23)
Effectiveness	23 (8.5)	26 (10)	34 (8)	15 (14.15)	11 (7.14)	8 (4.9)
Efficiency	20 (7.5)	45 (17.3)	53 (12.5)	12 (11.35)	33 (21.43)	8 (4.9)
Learnability	38 (14.12)	26 (10)	53 (12.5)	11 (10.4)	15 (9.75)	27 (16.57)
Error	68 (25.28)	50 (19.24)	93 (22)	25 (23.6)	25 (16.23)	43 (26.4)
No. of usability problems	269	260	423	106	154	163
Percentage of the problems identified by the two methods to the total number of problems				25%	36%	39%

Based on the results of the Chi-square test, a significant difference was observed between the numbers of problems identified by the two methods (*P* ≤ 0.0001). Furthermore, a significant difference was found between the number of problems identified by both methods in terms of usability attributes (i.e., *P* < 0.0001, *P* = 0.034, *P* < 0.0001, *P* < 0.0001, and *P* < 0.0001 for satisfaction, effectiveness, efficiency, learnability, and error, respectively). Moreover, from the total number of the problems i.e., 423 in the combined method (TA + HE), 39, 36, and 25% were detected by HE, TA, and both methods (TA&HE), respectively (Table 2).

Table 3 presents the mean severity level of problems identified by each of the methods per five usability attributes. Based on the results, the mean severity level of problems detected by both methods and the combined method was at the “Major” level (i.e., 3.34, 3.25, and 3.26 in TA, HE, and (TA + HE) methods, respectively).

**Table 3:** Mean and Standard Deviation of the severity scores of identified problems per method and usability attribute

	Heuristic evaluation, Mean ± SD	Think aloud, Mean ± SD	Think aloud + Heuristic evaluation, Mean ± SD	Both Think aloud and Heuristic evaluation, Mean ± SD	Only Think aloud, Mean ± SD	Only Heuristic evaluation, Mean ± SD
Satisfaction	3.17 ± 3.02	3.29 ± 0.36	3.22 ± 2.41	4.21 ± 4.83	3.29 ± 0.36	2.59 ± 0.65
Effectiveness	3.47 ± 0.59	3.65 ± 0.41	3.53 ± 0.55	3.79 ± 0.32	3.65 ± 0.46	2.85 ± 0.49
Efficiency	3.58 ± 0.66	3.31 ± 0.35	3.41 ± 0.5	3.96 ± 0.1	3.31 ± 0.35	2.92 ± 0.71
Learnability	3.05 ± 0.66	3.23 ± 0.21	3.1 ± 0.57	3.53 ± 0.59	3.23 ± 0.21	2.86 ± 0.59
Error	3 ± 0.67	3.22 ± 0.38	3.06 ± 0.61	3.55 ± 0.49	3.22 ± 0.38	2.67 ± 0.54
severity rating of Total	3.25 ± 0.25	3.34 ± 0.17	3.26 ± 1.67			

Generally, the result of the Mann-Whitney U test indicated no significant difference was found between the mean severity of problems identified by the two methods in terms of effectiveness (*P* = 0.44), learnability (*P* = 0.41), and error (*P* = 0.11) attributes. Accordingly, no significant difference was observed between the mean severities of problems detected by using the two methods (*P* = 0.43). However, a significant difference was found between the mean severities of problems identified by the two methods related to satisfaction and efficiency attributes (*P* = 0.001 and *P* = 0.01).

Additionally, Table 4 summarizes some of the most important problems identified by TA and HE. These problems were categorized in terms of the usability attributes.

**Table 4:** Problems detected by the two methods in terms of usability attributes

**Satisfaction**	**HE**	**TA**
The use of different colors for buttons, text fields, and links;	**+**	
The emergence of problems such as the lack of page heading and the invisibility of the actions;		**+**
The existence of redundant checkboxes, icons, and text fields;	**+**	
The presence of crowded system pages and the inappropriate division of all pages into three separate panels;		**+**
A failure to design the main menu as dropdown menu buttons;		**+**
The inappropriate layout and design of the tables, especially when they are empty;	**+**	
The display of two or more items concerning patient information in a text field (e.g., a patient’s name and code) and the possibility of moving the pointer in that text field;	**+**	
A failure in specifying the default option for radio buttons;	**+**	
The inaccessibility of the required information for nurses on the system pages (e.g., patients’ name, diagnosis and problem, medical history, and blood group);		**+**
The application of inappropriate colors for page backgrounds;		**+**
The use of inappropriate colors for the fonts like red and the small font sizes of the buttons;		**+**
**Effectiveness**	**HE**	**TA**
The inappropriate function of the buttons (e.g., a button called “New” fails to remove all fields by a single click);	**+**	
The use of a different language for tooltips of icons and buttons;	**+**	
The impossibility of creating reports at desired times;	**+**	
The impossibility of printing some reports;	**+**	
The lack of a breadcrumb element to display different steps for completing the tasks;		**+**
The inappropriate location and title of operational buttons;		**+**
The lack of feedback presentation regarding the users’ activities;		**+**
The inaccessible and inefficient search interface of the system;		**+**
The lack of any indication to push a required functional button such as “hospitalization order” in inpatient admission system;		**+**
The use of inappropriate labels of “outpatient admission” for the button designed for the referral of a patient from an emergency room to an inpatient ward;		**+**
**Efficiency**	**HE**	**TA**
Inability to print or exit by clicking on the “Print” button in some pages;	**+**	
A need for pushing the “Backspace” button to be able to exit from some system sections;	**+**	
The movement to another page instead of the previous page when pushing the “Undo” button;	**+**	
The lack of ability to close some windows;	**+**	
The lack of permission to search for the patients by the home screen of the inpatient admission system;		**+**
The presence of efficiency compromising problems such as the need for scrolling, navigating different pages, and taking various steps to perform a task;		**+**
The lack of a button for returning to the home page and patient information page;		**+**
The poor design of data entry fields such as failure to show the first data entry field by blinking cursor and the lack of distinction between the required and optional fields;		**+**
The lack of an interface for searching medication orders, laboratory test requests, and para-clinical procedures in the nursing information system;		**+**
A need for regular switching between mouse clicks and keystrokes to enter the data;		**+**
**Learnability**	**HE**	**TA**
The poor design and unclear functioning of some components such as checkboxes;	**+**	
The inappropriate shape of some icons;	**+**	
The use of similar icons for different tasks, as well as different icons for similar tasks;	**+**	
The display of user login information in unrelated fields;	**+**	
The lack of labels for text fields and checkboxes or the display of labels only in tooltips;	**+**	
Inappropriate and incomprehensible label for operating buttons such as “Close referral” and “Create referral”;		**+**
The ambiguous layout of the main menu options in the nursing information system;		**+**
Inappropriate labels for the subcategories of the main menu;		**+**
A need for remembering information from a location to another location due to the information dispersion and the lack of separation between physicians and nurses information;		**+**
The lack of a system help for the users;		**+**
**Error**	**HE**	**TA**
The lack of an error message when typing unauthorized characters in most entry text fields;	**+**	
The lack of Inline Validation of Data Entry Forms;	**+**	
The display of redundant error messages if clicked or right-clicked on some items;	**+**	
The lack of an error message when entering the wrong information in some fields, instead, the system completely hangs and fails;	**+**	
The possibility of searching with blank fields and the unauthorized change of patients information without receiving any error message;	**+**	
The demonstration of inappropriate error messages in response to the users’ mistakes;	**+**	
The use of varied colors for hyperlinks;	**+**	
The impossibility of easy error fixation (e.g., the cursor does not blink in the incomplete fields);		**+**
The induction for entering information in fixed fields due to using inappropriate colors for these fields;		**+**
The use of similar colors for distinct buttons, which leads to the impression of a relationship between these buttons;		**+**
The display of the wrong message like “*No patient is registered with this information*” instead of “*This patient had no previous encounter*”;		**+**

## Discussion

The results of the present study demonstrated that the number of the problems identified by the Think aloud (TA) and Heuristic Evaluation (HE) methods were different. In addition, both methods identified various problems related to each of the five usability attributes. Further, the mean severity of the problems identified by both methods was at the “Major” level and no significant difference was detected between the mean severities of the problems identified by these methods. However, merely a significant difference was observed between the mean severities of the problems related to the satisfaction and efficiency usability attributes.Consistent with the results of the studies by Karat [7] and Jeffries [8], in this study, HE significantly identified a higher number of problems compared to TA. Conversely, in two previous studies which compared the effectiveness of TA with Cognitive walkthrough (CW) [13], and HE with CW [27], no significant difference was found between the number of problems identified by each of these two methods. Contrary to the study by Hasan [28], in which HE and TA methods identified a higher number of “Minor” and “Major” usability problems, respectively, in the present study, the mean severity of problems identified by both methods was at the “Major” level. Similarly, Khajouei [13] reported that there was no significant difference between the mean severity scores of the problems identified by the two methods. Based on the results of the current study, a significant difference was detected between the two methods in terms of the number of problems identified related to each usability attribute. The TA method identified more problems concerning the effectiveness and efficiency attributes while more problems related to the satisfaction, learnability, and error attributes were identified by using the HE method. In a previous study [27], HE identified a higher number of problems related to satisfaction attribute as compared to CW. HE identified problems with “Major” and “Catastrophe” severity such as the inconsistency of button, fields, and the color of links; the use of the same icons for different tasks and vice versa; and system failure to respond when entering wrong information while TA falls short in finding these problems. Furthermore, TA identified high severity problems such as the need to take multiple steps to perform a task, and the lack of a feedback in response to the users’ actions as well as a search field. Using only HE results in missing such important problems.

TA mostly identified interactive problems which users encounter during the completion of tasks while HE missed these problems. Consistent with the study by Doubleday [29], in this study, each of the HE and TA methods identified many distinctive problems which were not identified by the other method. Based on these results, using only one of these methods in the development process of a system is unable to guarantee a complete usability of that system. Therefore, it is recommended combining these two methods to identify all types of problems and to improve the usability of the system.The results of this study highlighted that the HE method mostly identifies problems concerning inappropriate design of the user interface components. In line with this finding, the results of a previous study reported that this method often identifies common and general problems in the design of system user interfaces [9]. However, the TA method identifies problems which hinder users from accomplishing specific tasks due to the lack of some necessary features in the system. Examples of these problems are the impossibility of searching patients on the home page in the inpatient admission system, the lack of the functionality to retrieve laboratory tests and medications in the nursing information system, failure to display information needed by clinicians, the lack of system help and a breadcrumb element, and failure to provide feedback in response to users’ actions. Given that none of the HE principles cover these problems, results of HE may not fully meet the cognitive needs of users. By considering the limited scope of problems identified by each method, it is recommended to apply a combination of the two methods (TA + HE) to effectively evaluate a health information system.

Our work clearly has some limitations. First, evaluating all modules of the Social Security Electronic System was impossible since this Hospital Information System is a large system with multiple modules. In this regard, to be able to examine the maximum functionalities of the system, we evaluated two clinical and administrative modules of this system (i.e., nursing information system and inpatient admission information system). Second, to avoid interference with providing health care services to patients and adhere to the regulations of patient safety, the usability tests were performed in the laboratory setting. To simulate the real working environment without threatening patients’ safety we used dummy patient information. In addition, the scenarios were designed in such a way that they cover all real user tasks, including simple, medium, and complex tasks. Finally, since the users only could accomplish each task once, it was impossible to examine potential problems related to the memorability attribute. Accordingly, users emphasized their need for training to learn how to use the system effortlessly and sought for the system help. These results indicate potential memorability problems of the systems. Future studies can identify memorability problems by conducting the tests at appropriate intervals.The previous studies [14, 30–34] that compared the effectiveness of one or both of the methods used in this study recruited a lower number of evaluators or users than the present study. These studies only evaluated a single system and did not use statistical analysis to compare the methods. Based on the results, there were significant differences between the two methods in terms of the number and type of usability problems. Consistent with the results of previous studies [14, 30, 35, 36], the results of this study emphasize using a combination of the two methods as complementary to each other. The results of the present study can help the decision-makers and information technology managers of hospitals and clinical centers to select an appropriate method for evaluating the usability of health information systems and to improve it. As a result, the end-users of these systems, especially nurses and physicians will have an easy and successful interaction with these systems.

## Conclusion

The results demonstrated that each of think aloud (TA) and heuristic evaluation (HE) methods can identify different usability problems. The HE method mostly detected problems related to satisfaction, learnability, and error prevention attributes while the TA method mainly identified problems related to effectiveness and efficiency attributes. Since the problems detected by each of the methods were at a “Major” severity level, using only one of these methods can result in missing a number of important problems which are merely detectable by the other method. Since using a combination of user-based and expert-based methods can lead to the identification of almost all the usability problems, it is recommended to use it for evaluating the usability of healthcare information systems. In the present study, we combined two of the most common user-based and expert-based methods. Since there are various methods of user-based and expert-based methods, future studies can examine the effect of combining other methods. This can provide a good insight for selecting the most appropriate method to evaluate specific systems.

## Data Availability

The data generated and analyzed during this study are available from the corresponding author on reasonable request.

## Abbreviations

TA: Think aloud
HE: Heuristic evaluation
SSES: Social security electronic system
SSO: Social Security Organization
ICU: Intensive care unit
CW: Cognitive walkthrough
ISO: International standard organization
